# The Crystal Structure of a hCA VII Variant Provides Insights into the Molecular Determinants Responsible for Its Catalytic Behavior

**DOI:** 10.3390/ijms19061571

**Published:** 2018-05-24

**Authors:** Martina Buonanno, Anna Di Fiore, Emma Langella, Katia D’Ambrosio, Claudiu T. Supuran, Simona Maria Monti, Giuseppina De Simone

**Affiliations:** 1 Istituto di Biostrutture e Bioimmagini, CNR, Via Mezzocannone 16, 80134 Napoli, Italy; martinabuonanno@gmail.com (M.B.); anna.difiore@cnr.it (A.D.F.); emma.langella@cnr.it (E.L.); Katia.dambrosio@cnr.it (K.D.); 2 Dipartimento Neurofarba, Sezione di Scienze Farmaceutiche e Nutraceutiche, Università degli Studi di Firenze, Via U. Schiff 6, 50019 Florence, Italy; claudiu.supuran@unifi.it

**Keywords:** carbonic anhydrases, water network, proton transfer, proton shuttle, catalytic efficiency, pK_a_

## Abstract

Although important progress has been achieved in understanding the catalytic mechanism of Carbonic Anhydrases, a detailed picture of all factors influencing the catalytic efficiency of the various human isoforms is still missing. In this paper we report a detailed structural study and theoretical pKa calculations on a hCA VII variant. The obtained data were compared with those already known for another thoroughly investigated cytosolic isoform, hCA II. Our structural studies show that in hCA VII the network of ordered water molecules, which connects the zinc bound solvent molecule to the proton shuttle His64, is altered compared to hCA II, causing a reduction of the catalytic efficiency. Theoretical calculations suggest that changes in solvent network are related to the difference in pKa of the proton shuttle in the two enzymes. The residue that plays a major role in determining the diverse pKa values of the proton shuttle is the one in position four, namely His for hCA II and Gly for hCA VII. This residue is located on the protein surface, outside of the active site cavity. These findings are in agreement with our previous studies that highlighted the importance of histidines on the protein surface of hCA II (among which His4) as crucial residues for the high catalytic efficiency of this isoform.

## 1. Introduction

Carbonic Anhydrases (CAs, EC 4.2.1.1) are ubiquitous metallo-enzymes that are present in most living organisms [1]. Seven genetically distinct families have been identified so far: the α-, β-, γ-, δ-, ζ-, η-, and θ-CAs [1,2,3,4,5,6,7,8]. Human CAs (hCAs) belong to the α-class and exist in fifteen isoforms. Although sharing a high level of three-dimensional similarity and a zinc ion in the active site, these isoforms differ in the tissue distribution, catalytic activity and cellular localization. Indeed, five isoforms are cytosolic (CAs I-III, VII and XIII), four are membrane-associated (CAs IV, IX, XII and XIV), two are mitochondrial (CAs VA and VB), and one is a secretory protein present in milk and saliva (CA VI) [1,9,10].

hCAs catalyze a simple but fundamental physiological reaction, the reversible hydration of CO_2_ to HCO_3_^−^ and proton, following a two-step mechanism described by Equations (1) and (2) [1]. The first step consists of the nucleophilic attack of the Zn^2+^-bound hydroxide on CO_2_ with consequent formation of HCO_3_^−^. The binding of HCO_3_^−^ to the zinc is rather labile, thus it is replaced by a water molecule generating the catalytically inactive form of the enzyme EZn^2+^–H_2_O (Equation (1)). In the second step of the reaction, which is the rate-limiting one, the zinc-bound hydroxide is regenerated through a proton transfer reaction from the zinc-coordinated water molecule to the bulk solvent (B) [11,12,13,14].

(1) $${\mathrm{EZn}}^{2+}–{\mathrm{OH}}^{-}+{\mathrm{CO}}_{2}⇆{\mathrm{EZn}}^{2+}–{{\mathrm{HCO}}_{3}}^{-}\overset{H_{2}O}{⇆}{\mathrm{EZn}}^{2+}–H_{2}O+{{\mathrm{HCO}}_{3}}^{-}$$

EZn^2+^–H_2_O + B ⇆ EZn^2+^–OH^−^ + BH^+^ (2)

In the majority of the isoforms, a histidine residue placed in the middle of the active site cavity, namely His64, assists this step acting as a proton shuttle [15,16]. Accordingly, isoforms lacking a histidine in position 64 generally show a lower catalytic efficiency [17]. Moreover, the replacement of His64 with Ala in hCA II results in a 10–50-fold decrease of catalysis compared with the wild type enzyme [16,18].

**Figure 1:**
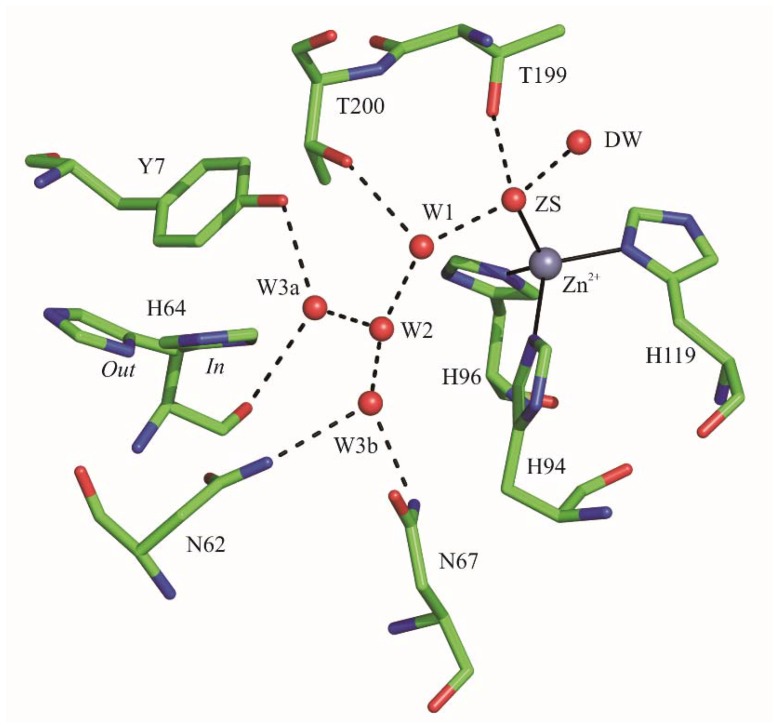
Active site of hCA II (PDB accession code 1TE3) [20] showing the well-ordered water molecules that connect the Zn^2+^-bound solvent molecule (ZS) to the proton shuttle residue. His64 is present both in its *in* and *out* conformation. The solvent molecules are named as reported by Fisher et al., 2005 [20]. The zinc ion coordination and the deep water (DW) are also depicted. Hydrogen bonds are reported as dashed lines.

Currently, most of the studies on the catalytic mechanism of hCAs have been performed on the ubiquitous isoform hCA II, which is the most active isozyme. Results demonstrated that the His64 side chain has two conformational states, named *in* and *out*. In the *in* conformation, the imidazole ring points toward the zinc ion, whereas in the *out* conformation it points out of the active site cavity [19,20]. Interestingly, even if it was initially suggested that the His64 conformational mobility was an essential requirement for proton transfer [11], more recent studies have questioned its necessity [14,21]. Apart from the presence of His64, other structural features have been recognized as important factors affecting the rate-limiting step of the catalytic reaction [22,23,24,25]. In particular, in hCA II the presence of a network of well-ordered water molecules, connecting the Zn^2+^-bound solvent molecule to His64 (see Figure 1), finely modulates the efficiency of proton transfer during catalysis [14]. Accordingly, site directed mutagenesis of several hCA II residues (Tyr7, Ans62, Asn67, Thr199, and Thr200), which have been shown to interact with these water molecules, caused changes in solvent positions and consequently in the rate of the proton transfer process [26,27,28,29].

Among the cytosolic isoforms, we recently focused our attention on hCA VII [30,31,32,33,34,35], an enzyme initially detected only in brain, where it functionally participates as a molecular switch for GABAergic excitation [30,36,37]. Subsequently, hCA VII was found also in other tissues, such as colon, muscle, and liver [38]. hCA VII has been proposed as a target for CA inhibitors to be used for treatment of epilepsy [37] and chronic neuropathic pain [39]. Recent studies from our group also unveiled for this enzyme a potential role as an oxygen radical scavenger, protecting cells from oxidative damage [33].

hCA VII has been fully characterized from both structural and catalytic viewpoints [32,35]. In particular, crystallographic studies performed on a mutated form of the enzyme in complex with the sulfonamide inhibitor acetazolamide showed that hCA VII structure consists of a central ten-stranded β-sheet surrounded by several helices and additional β-strands, as observed for other hCAs [1]. The active site is located in a conical cavity, with the catalytic zinc ion at the bottom [32].

Catalytic assays demonstrated that this enzyme is able to catalyze the CO_2_ hydration reaction with slightly less efficiency compared to the cytosolic isoform hCA II (hCA VII: k_cat_ = 7.9 × 10^5^ s^−1^ and k_cat_/K_M_ = 7.2 × 10^7^ M^−1^ s^−1^; hCA II: k_cat_ = 1.4 × 10^6^ s^−1^ and k_cat_/K_M_ = 1.5 × 10^8^ M^−1^ s^−1^) [35]. This finding was quite surprising considering the high degree of sequence and structural similarity between hCA II and hCA VII. Thus, with the aim to understand the structural factors responsible for the lower catalytic efficiency of hCA VII with respect to hCA II, we performed a crystallographic study of an unbound variant of hCA VII and compared the obtained structure with that of the native hCA II. Theoretical pK_a_ calculations of the proton shuttle His64 were also carried out.

## 2. Results

### 2.1. Crystallographic Studies

In a previous study, we reported the bacterial expression, using pGex-6P-1 vector, of a mutated form of hCA VII, named dmCA VII, which contained two amino acid substitutions. In particular, the cysteine residues in position 183 and 217 were mutated to serines. It was demonstrated that the two mutations did not influence the catalytic activity of the enzyme; indeed, the kinetic constants for native hCA VII and dmCA VII were absolutely comparable (dmCA VII: k_cat_ = 7.0 × 10^5^ s^−1^ and k_cat_/K_M_ = 6.5 × 10^7^ M^−1^ s^−1^) [35]. dmCA VII was then used for structural studies since it avoided potential crystallization problems caused by the mixture of reduced and oxidized enzyme forms [32].

In this paper, dmCA VII was produced using a different vector, pETM13, which allowed us to successfully express the protein with a C-terminal histidine tag in the soluble fraction of *E. coli*. The purification steps led to obtain the protein with high purity level (>98%) and a final yield of 7 mg/L. The enzyme was concentrated at 5 mg/mL and crystallized, using polyethylene glycol as precipitant, in the space group P2_1_2_1_2 with one molecule per asymmetric unit. Using the tagged protein for crystallization experiments, more reproducible results were obtained in comparison with the same trials performed on the protein expressed in the pGex-6P-1 vector.

The structure was determined at 1.91 Å resolution and atomic coordinates were deposited in the Protein Data Bank (PDB) as entry 6G4T. The crystal parameters, data collection, and refinement statistics are listed in Table 1.

**Figure 2:**
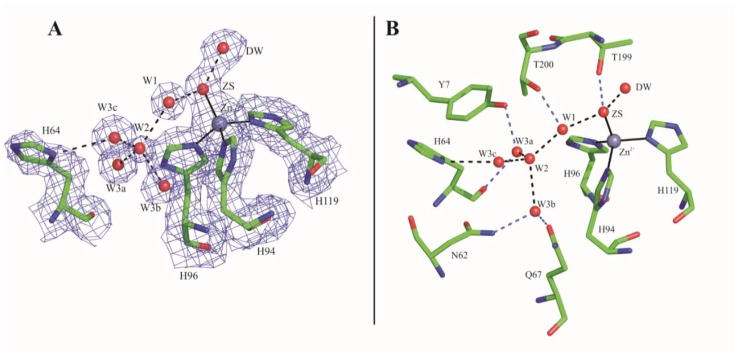
(**A**) Active site region of dmCA VII, here determined, with associated sigma-A weighted 2|Fo|-|Fc| electron density map contoured at 1.0 σ. The map clearly indicates a unique conformation for His64. (**B**) Representation of the hydrogen bond network, which connects the Zn^2+^-bound solvent molecule (ZS) to the proton shuttle. The zinc ion coordination and the deep water (DW) are also reported.

The comparative analysis between the obtained structure and the one with the same enzyme crystallized in complex with acetazolamide did not reveal any significant differences [32]. Indeed, the superposition of corresponding Cα atoms between the two enzymes leads to an r.m.s.d. of 0.32 Å, indicating that the presence of a ligand in the active site did not alter the backbone conformation. The conformation of residues delimiting the active site cavity was also conserved. Interestingly, the proton shuttle His64 was modelled in both structures only in the *out* conformation; however, in the structure under investigation no evidence of the inward conformation was observed (Figure 2A), whereas some disorder was observed in the case of the inhibited enzyme [32].

In the structure of the unbound dmCA VII, the metal ion maintains the tetrahedral coordination already reported for the inhibited enzyme; in this case the fourth coordination position is occupied by a solvent molecule (ZS), which is connected to His64 through a network of well-ordered water molecules (Figure 2A,B). In detail, ZS is at hydrogen bond distance from the deep water (DW, the solvent molecule that occupies the CO_2_ binding pocket) and from the water molecule W1, which in turn interacts with W2 (nomenclature of water molecules refers to that adopted for hCA II by Fisher et al. 2005) [20]. The latter is connected with three other water molecules (W3a-c), W3a is hydrogen bonded with the carbonyl oxygen of His64 and the side chain of Tyr7, W3b interacts with Asn62 and Gln67 side chains, and W3c is at 3.2 Å from the ND1 atom of His64.

**Figure 3:**
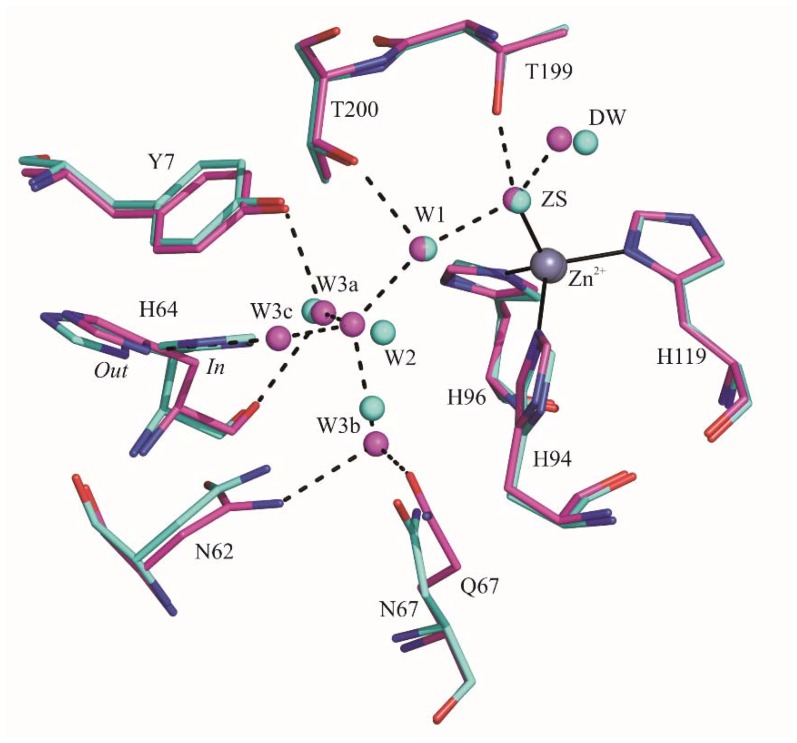
Structural superposition of dmCA VII (colored in magenta) and hCA II (colored in cyan) active site. Hbonds present in dmCA VII are shown as dashed lines.

A comparative analysis between unbound dmCA VII and native hCA II revealed that the amino acids present within the active site cavity adopt a generally conserved conformation. The most significant difference was observed for the proton shuttle residue which, differently from dmCA VII, in hCA II adopts both the *in* and the *out* conformations (Figure 3) [20,40,41,42]. The network of water molecules, that connects ZS to His64 in both enzymes, also reveals a substantial similarity (Figure 3). The unique significant difference is that in the case of dmCA VII an additional water molecule is present, namely W3c, which is located in the same position that in hCA II is occupied by His64 side chain in the *in* conformation. This molecule is hydrogen bonded to the His64 side chain (Figure 2B).

### 2.2. pK_a_ Calculations

Several papers highlighted a correlation between the pK_a_ value of hCA II His64 and conformational preferences of its side chain (see Discussion) [26,29]. Thus, we carried out theoretical predictions of His64 pK_a_ value in dmCA VII as well as in hCA II using the PROPKA method [43,44]. Both *in* and *out* conformations of His64 present in the native hCA II crystal structure (PDB code 1TE3) [20] were taken into account for calculations. In the case of dmCA VII structure, which shows only His64 *out* conformation, a model of the *in* conformation was built using the hCA II structure as template [20].

Predicted pK_a_ values are shown in Table 2. These values are significantly lower than those experimentally determined for hCA II His64 [20]. This finding is not surprising, since it is reported in literature [44,45] an underestimation of protein stabilizing interactions for buried residues in PROPKA calculations, which leads to a pK_a_ lowering. This is the case of His64 in CA structures, which is 63% buried in the *out* conformation and 100% buried in the *in* conformation. However, since this effect is present in a comparable manner in all structures under investigation, it is possible to make a relative comparison and to understand the effect of the chemical environment in modulating His64 pK_a_ in hCA VII and hCA II.

**Figure 4:**
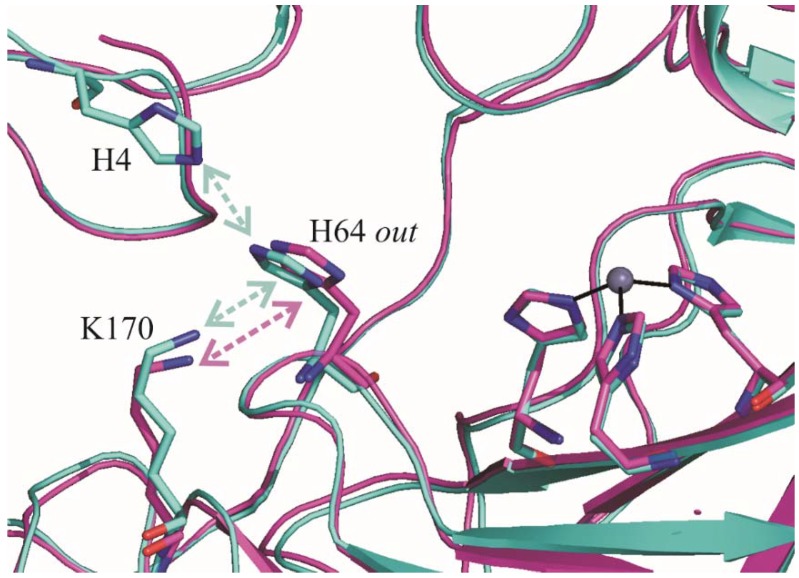
Structural comparison of His64 environment in dmCA VII (magenta) and hCA II (cyan). Residues affecting the pK_a_ of His64 *out* conformation are in sticks and indicated by arrows. The catalytic triad and the zinc ion are also depicted.

These calculations reveal that the pK_a_ value of His64 is higher in dmCA VII, for both *in* and *out* conformations (Table 2). Moreover, for both enzymes, the *out* conformation displays a higher pK_a_ value than the *in* conformation. Indeed, the His64 *in* conformation, pointing toward the active site interior, is completely hindered from solvent (100% buried) and feels a slight destabilizing charge-charge interaction with Zn^2+^ ion that further lowers its pK_a_ value. On the contrary, the pK_a_ of the *out* conformation in both enzymes is affected by the presence of Lys170, a residue located on the protein surface at the top of the active site cavity (Figure 4).

In the case of hCA II, there is an additional residue, i.e., His4, which further affects the pK_a_ value of the *out* conformation (Figure 4). Interestingly, this residue is substituted by a Gly in dmCA VII. His4 residue is predicted by PROPKA calculations to titrate at pH 6.3 (pK_a_ = 6.3), being completely solvent exposed. The electrostatic interaction between His4 and the nearby His64 residue contributes to hamper the titration of His64 and thus to lower its pK_a_ in hCA II. This effect is, however, strongly dependent on the distance between His4 and His64. Since from the analysis of the hCA II structures crystallized in different conditions [20,40,42], it emerged that the side chain of His4 can assume different orientations, we decided to perform pK_a_ calculations on different hCA II structures (see Method paragraph) (Table 3). The final pK_a_ value of hCA II His64 in the *out* conformation (Table 2) was obtained by averaging over the values reported in Table 3, and further confirms that the pK_a_ of His64 is higher in dmCA VII (4.6) than in hCA II (4.3).

## 3. Discussion

The aim of this work was to provide insights into the molecular features responsible for the different catalytic activity of hCA II and hCA VII. Two features of these enzymes were thoroughly analyzed and compared: (i) the network of ordered water molecules, connecting the zinc bound solvent molecule and the proton shuttle His64, and (ii) the pK_a_ of this residue. Indeed, in previous studies these two features were reported to be important in determining the rate of the proton transfer [14].

**Figure 5:**
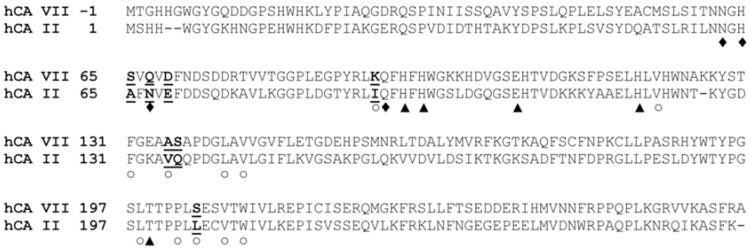
Sequence alignment of hCA VII and hCA II. The catalytic histidines, Thr199 and Glu106 are indicated with a triangle, residues belonging to the hydrophobic region of the active site are indicated with a rhombus, while residues belonging to the hydrophilic one are indicate with a circle. Finally, residues of the active site cavity which are not conserved in the two enzymes are underlined in bold style.

hCA II and hCA VII present a high degree of sequence identity (55.7%), which is mainly localized in the active site region. Indeed, only seven substitutions (S65A, Q67N, D69E, K91I, A135V, S136Q and S204L) are observed among the 23 residues that delimit the active site cavity (Figure 5). Accordingly, the structural superposition of the unbound hCA VII and the native hCA II, crystallized at comparable pH values (PDB codes 1TEQ and 1TE3) [20], reveals a substantial degree of three-dimensional similarity in the conformation of conserved residues belonging to the active sites of the two enzymes. The only notable difference is related to the conformation of His64. Indeed, this residue presents in hCA II both the *in* and the *out* conformations, whereas only the *out* conformation is observed for dmCA VII.

The observation that in dmCA VII only the *out* conformation is present is quite surprising. Indeed, as mentioned in the Introduction, in many crystallographic structures of hCA II [20,40,41,42] this residue shows high conformational flexibility and is observed both in the *in* and *out* conformation [19,20]. The two conformational states are approximately equally populated at physiological pH, but a predominance of *in* conformation is observed at more alkaline pH [19,20]. Since dmCA VII has been crystallized at pH 8.5, in agreement with what was observed for hCA II, a predominant *in* conformation would have been expected, differently from what experimentally observed. A predominant *out* conformation of His64 was observed also in the hCA II variants N67L [26], T200S [21], W5H [46], W5E [46], N67Q [47] and N62D [29]. For some of these variants a clear correlation between a higher pK_a_ of this residue and the *out* conformation was observed [26,29]. When this correlation was not present, stabilizing interactions of the imidazole ring (including crystal packing interactions) were utilized to justify the observed *out* conformation [46]. In agreement with data showing the correlation between the *out* conformation of His64 and its higher pK_a_ values, our theoretical calculations demonstrated that the pK_a_ of His64 is higher in dmCA VII than in hCA II.

The different conformational preferences of His64 in hCA II and dmCA VII have important consequences in the solvent structure within the active site cavity. Indeed, the structural comparison between the two enzymes reveals that in the case of dmCA VII an additional water molecule connecting the zinc-bound solvent molecule and His64 is present in the position that in hCA II is occupied by the *in* conformation of His64. Previous studies reported by Fisher and coworkers [20] proposed that the efficiency of proton transfer is related to the number of water molecules between the Zn^2+^-bound solvent molecule and the proton shuttle: the smaller the number of connecting water molecules is, the higher the catalytic efficiency will be. These observations were in contrast with the work of Riccardi et al. [48], which suggested that the intramolecular proton transfer is governed by electrostatics and is not sensitive to distance nor number of water molecules bridging the zinc-water and the proton shuttle. Our findings are in agreement with the hypothesis proposed by Fisher. In fact, in our case the higher number of intervening water molecules is associated to a reduced catalytic efficiency of hCA VII with respect to hCA II.

In conclusion, our studies provide a further piece in the complicated puzzle of the hCA catalytic mechanism. In particular, we found that in hCA VII pKa of His64 is higher than in hCA II. This determines a predominant *out* conformation of its side chain and consequently an altered solvent structure that makes less efficient the catalytic mechanism. Interestingly, a key residue in determining the different pKa values of the proton shuttle in the two enzymes, is the one in position four, namely His4 for hCA II and Gly4 for hCA VII. Previously reported studies from our group highlighted the crucial role of histidines on the protein surface of hCA II (among which His4) as important in determining the high catalytic efficiency of this isoform [45,46]. The studies here reported corroborate this hypothesis; mutagenesis experiments are currently underway in our lab to definitively confirm it.

## 4. Materials and Methods

### 4.1. Protein Expression and Purification

The cDNA encoding the C183S/C217S mutant form of hCA VII, called dmCA VII [32], was PCR amplified using the following site-specific primers with NcoI/XhoI restriction sites:

- F: 5′-CGCGCGCCATGGGCATGACCGGCCACCACG-3′
- R: 5′-CGCGCGCTCGAGGGCCCGGAAGGAGGC-3′

The resulting fragment was ligated into the expression vector pETM13 (a kind gift from EMBL, Heidelberg). The generated plasmid was checked by sequencing and appropriate digestion with restriction enzymes. *E. coli* BL21 (DE3) cells were transformed with the recombinant construct. The growth was performed at 37 °C. At OD_600_ = 0.6, cells were induced by adding IPTG (isopropil-β-d-1-tiogalactopyranoside) at a final concentration of 0.1 mM. The expression of the recombinant protein was carried out at 22 °C for 16 h. The culture was centrifuged (20 min at 4 °C at 27,956× *g*) and the pellet stored at −80 °C.

The lysis of the obtained pellet was performed in PBS 1X (10 mM phosphate buffer, 27 mM potassium chloride and 137 mM sodium chloride, pH 7.4), in presence of 1 mM phenylmethanesulfonyl fluoride, 5 mg/mL DNaseI, 0.1 mg/mL lysozyme and 1.0 µg/mL Aprotinin, Pepstatin and Leupeptin used as protease inhibitors (Apllichem, Darmstadt, Germany). Cells were disrupted by sonication. After centrifugation (30 min at 4 °C at 219,126× *g*), the protein was purified by FPLC, using an AKTA system on a 1 mL His Trap FF column (GE Healthcare, Little Chalfont, UK), by stepwise elution, according to the manufacturer’s instruction (GE Healthcare, Little Chalfont, UK). After elution, dmCA VII enzyme was dialyzed in 20 mM Tris, 100 mM NaCl, pH 8.0 buffer. The protein was further purified by affinity chromatography with p-(aminomethyl) benzene sulfonamide (pAMBS) agarose beads (Sigma, Milan, Italy). Protein purity was assessed on 15% SDS-PAGE gels, using Biorad Precision Plus Protein All Blue Standards (10–250 kDa) as molecular mass marker.

### 4.2. Crystallization and X-ray Data Collection

hCA VII crystallization conditions were identified using Crystal Screen, Crystal Screen 2 and Index kits from Hampton Research [49,50]. The wells contained 500 μL precipitant solution and the drops were composed of 1 μL reservoir solution and 1 μL protein solution at a concentration of 5 mg/mL in 20 mM Tris-HCl, pH 8.0, 150 mM NaCl. Crystals suitable for X-ray analysis were obtained using 25% (*w*/*v*) polyethylene glycol 3350 in 0.1 M Tris-HCl, pH 8.5 as precipitant buffer. Crystals appeared in the drops within 48 h and grew in about 1 week to maximum dimensions of 0.2 × 0.2 × 0.2 mm^3^. A complete dataset was collected at 1.91 Å resolution from a single crystal at the temperature of 100 K, using a copper rotating-anode generator developed by Rigaku equipped with a Rigaku Saturn CCD detector. Prior to cryogenic freezing, crystals were transferred to the precipitant solution with the addition of 25% (*w*/*v*) glycerol. Diffraction data were indexed, integrated and scaled using the HKL2000 software package [51]. Crystals belonged to the space group P2_1_2_1_2 with unit cell dimensions of a = 66.3 Å, b = 89.4 Å, c = 44.4 Å and one molecule for asymmetric unit. Data-collection statistics are reported in Table 1.

### 4.3. Structure Determination and Refinement

The structure of dmCA VII was solved by the molecular replacement technique using the program AMoRe [52] and the atomic coordinates of dmCA VII crystallographic structure in complex with acetazolamide inhibitor (PDB code 3ML5) [32] as a search model. The rotation and translation functions were calculated using data between 15.0 and 3.5 Å resolution. The one body translation search, using the centered-overlap function (c-o), on the first 50 rotation solutions led to a single solution with a correlation coefficient of 0.672 and an R-factor of 0.327. Refinement of the structure was performed with CNS 1.3 [53,54] and model building was performed with O [55]. Several cycles of manual modeling of the structure and positional and temperature factor refinement were carried out to reduce the crystallographic R-work and R-free values (in the 31.5–1.91 Å resolution range) to 0.196 and 0.244, respectively. The model stereochemistry was checked using the programs WHATCHECK and PROCHECK [56,57]. The final model contains 2063 protein atoms and 162 water molecules. Coordinates were deposited in the Protein Data Bank (PDB accession code 6G4T).

### 4.4. Theoretical pK_a_ Calcuations

pK_a_ values of His64 residue were predicted using PROPKA 3.0 (freely available at http://nbcr-222.ucsd.edu/pdb2pqr_2.0.0/) [44]. PROPKA is an empirical pK_a_ predicting method, which estimates the shift in the pK_a_ arising from hydrogen bonds, relative burial and coulombic interactions [43,44]. These contributions are parametrized to fit experimentally measured ΔpK_a_. Calculations were performed using the dmCA VII crystal structure herein reported, and all the available hCA II crystal structures collected in a pH range 7–9 (PDB accession codes 1TE3, 3KS3, 2CBA, 1TBT and 1TEQ) [20,40,42].

## Figures and Tables

**Table 1:** Data collection and refinement statistics.

Cell Parameter	Value
Space group	P2_1_2_1_2
Unit cell parameters (Å)	a = 66.3
	b = 89.4
	c = 44.4
Number of independent molecules	1
Data collection statistics	
Resolution limits (Å)	31.5–1.91
Wavelength (Å)	1.54178
Temperature (K)	100
Total reflections	94217
Unique reflections	20846
Redundancy	4.5
Completeness (%)	98.8 (87.3)
R-merge (%) *	0.079 (0.520)
<I>/<σ(I)>	14.7 (2.0)
Refinement statistics	
Resolution limits (Å)	31.5–1.91
R-work ** (%)	19.6
R-free ** (%)	24.4
r.m.s.d. from ideal geometry:	
Bond lengths (Å)	0.008
Bond angles (°)	1.4
Number of protein atoms	2063
Number of water molecules	162
Average B factor (Å2)	
All atoms	21.1
Protein atoms	20.7
Water molecules	25.6

* R-merge = Σ_hkl_Σ_i_|I_i_(hkl)-<I(hkl)>|/Σ_hkl_Σ_i_I_i_(hkl), where I_i_(hkl) is the intensity of an observation and <I(hkl)> is the mean value for its unique reflection; summations are over all reflections; ** R-work = Σ_hkl_||Fo(hkl)| − |Fc(hkl)||/Σ_hkl_|Fo(hkl)| calculated for the working set of reflections. R-free is calculated as for R-work, but from 5% of the data that was not used for refinement. Values in parentheses are referred to the highest resolution shell (1.94–1.91 Å).

**Table 2:** pK_a_ predictions of His64 *in* and *out* conformations for dmCA VII (this work) and hCA II (pdb code 1TE3) [20] enzymes. Calculations were performed using PROPKA 3.0 [43,44].

Enzyme	pK_a_	
	His64 *in*	His64 *out*
dmCA VII	3.8 *	4.6
hCA II	3.7	4.2 (4.3 ^#^)

* Model of His64 *in* conformation; ^#^ Mean value obtained by averaging over values listed in Table 3.

**Table 3:** pK_a_ predictions of His64 in *out* conformation using different crystal structures of hCA II [20,40,42]. A and B letters indicate two different conformers of His4 side-chain, when they are present in the same crystal structures.

hCA II Structures	pK_a_
	His64 *out*
1TE3	4.2
3KS3 (A)	4.3
3KS3 (B)	4.7
2CBA (A)	3.9
2CBA (B)	4.2
1TBT	4.3
1TEQ	4.4

## References

[1] Alterio V., Di Fiore A., D’Ambrosio K., Supuran C.T., De Simone G. (2012). Multiple binding modes of inhibitors to carbonic anhydrases: How to design specific drugs targeting 15 different isoforms?. Chem. Rev..

[2] Del Prete S., Vullo D., Fisher G.M., Andrews K.T., Poulsen S.A., Capasso C., Supuran C.T. (2014). Discovery of a new family of carbonic anhydrases in the malaria pathogen plasmodium falciparum—The eta-carbonic anhydrases. Bioorg. Med. Chem. Lett..

[3] Kikutani S., Nakajima K., Nagasato C., Tsuji Y., Miyatake A., Matsuda Y. (2016). Thylakoid luminal theta-carbonic anhydrase critical for growth and photosynthesis in the marine diatom phaeodactylum tricornutum. Proc. Natl. Acad. Sci. USA.

[4] Xu Y., Feng L., Jeffrey P.D., Shi Y., Morel F.M. (2008). Structure and metal exchange in the cadmium carbonic anhydrase of marine diatoms. Nature.

[5] Smith K.S., Jakubzick C., Whittam T.S., Ferry J.G. (1999). Carbonic anhydrase is an ancient enzyme widespread in prokaryotes. Proc. Natl. Acad. Sci. USA.

[6] Alterio V., Monti S.M., De Simone G., Frost S.C., McKenna R. (2014). Thermal-stable carbonic anhydrases: A structural overview. Carbonic Anhydrase: Mechanism, Regulation, Links to Disease, and Industrial Applications.

[7] Alterio V., Langella E., De Simone G., Monti S.M. (2015). Cadmium-containing carbonic anhydrase CDCA1 in marine diatom *Thalassiosira weissflogii*. Mar. Drugs.

[8] De Simone G., Di Fiore A., Capasso C., Supuran C.T. (2015). The zinc coordination pattern in the eta-carbonic anhydrase from plasmodium falciparum is different from all other carbonic anhydrase genetic families. Bioorg. Med. Chem. Lett..

[9] Supuran C.T. (2008). Carbonic anhydrases: Novel therapeutic applications for inhibitors and activators. Nat. Rev. Drug Discov..

[10] Supuran C.T., De Simone G. (2015). Carbonic Anhydrases as Biocatalysts. From Theory to Medical and Industrial Applications.

[11] Silverman D.N., McKenna R. (2007). Solvent-mediated proton transfer in catalysis by carbonic anhydrase. Acc. Chem. Res..

[12] Aggarwal M., Boone C.D., Kondeti B., McKenna R. (2013). Structural annotation of human carbonic anhydrases. J. Enzym. Inhib. Med. Chem..

[13] Boone C.D., Pinard M., McKenna R., Silverman D. (2014). Catalytic mechanism of alpha-class carbonic anhydrases: CO_2_ hydration and proton transfer. Subcell. Biochem..

[14] Mikulski R.L., Silverman D.N. (2010). Proton transfer in catalysis and the role of proton shuttles in carbonic anhydrase. Biochim. Biophys. Acta.

[15] Aggarwal M., Kondeti B., Tu C., Maupin C.M., Silverman D.N., McKenna R. (2014). Structural insight into activity enhancement and inhibition of H64A carbonic anhydrase II by imidazoles. IUCrJ.

[16] Tu C.K., Silverman D.N., Forsman C., Jonsson B.H., Lindskog S. (1989). Role of histidine 64 in the catalytic mechanism of human carbonic anhydrase II studied with a site-specific mutant. Biochemistry.

[17] Jewell D.A., Tu C.K., Paranawithana S.R., Tanhauser S.M., LoGrasso P.V., Laipis P.J., Silverman D.N. (1991). Enhancement of the catalytic properties of human carbonic anhydrase III by site-directed mutagenesis. Biochemistry.

[18] Duda D., Tu C., Qian M., Laipis P., Agbandje-McKenna M., Silverman D.N., McKenna R. (2001). Structural and kinetic analysis of the chemical rescue of the proton transfer function of carbonic anhydrase II. Biochemistry.

[19] Nair S.K., Christianson D.W. (1991). Structural properties of human carbonic anhydrase II at pH 9.5. Biochem. Biophys. Res. Commun..

[20] Fisher Z., Hernandez Prada J.A., Tu C., Duda D., Yoshioka C., An H., Govindasamy L., Silverman D.N., McKenna R. (2005). Structural and kinetic characterization of active-site histidine as a proton shuttle in catalysis by human carbonic anhydrase II. Biochemistry.

[21] Krebs J.F., Fierke C.A., Alexander R.S., Christianson D.W. (1991). Conformational mobility of His-64 in the Thr-200→Ser mutant of human carbonic anhydrase II. Biochemistry.

[22] Taraphder S., Maupin C.M., Swanson J.M., Voth G.A. (2016). Coupling protein dynamics with proton transport in human carbonic anhydrase II. J. Phys. Chem. B.

[23] Maupin C.M., Voth G.A. (2010). Proton transport in carbonic anhydrase: Insights from molecular simulation. Biochim. Biophys. Acta.

[24] Maupin C.M., McKenna R., Silverman D.N., Voth G.A. (2009). Elucidation of the proton transport mechanism in human carbonic anhydrase II. JACS.

[25] Kim C.U., Song H., Avvaru B.S., Gruner S.M., Park S., McKenna R. (2016). Tracking solvent and protein movement during CO_2_ release in carbonic anhydrase II crystals. Proc. Natl. Acad. Sci. USA.

[26] Fisher S.Z., Tu C., Bhatt D., Govindasamy L., Agbandje-McKenna M., McKenna R., Silverman D.N. (2007). Speeding up proton transfer in a fast enzyme: Kinetic and crystallographic studies on the effect of hydrophobic amino acid substitutions in the active site of human carbonic anhydrase II. Biochemistry.

[27] Domsic J.F., Williams W., Fisher S.Z., Tu C., Agbandje-McKenna M., Silverman D.N., McKenna R. (2010). Structural and kinetic study of the extended active site for proton transfer in human carbonic anhydrase II. Biochemistry.

[28] Huang S., Sjoblom B., Sauer-Eriksson A.E., Jonsson B.H. (2002). Organization of an efficient carbonic anhydrase: Implications for the mechanism based on structure-function studies of a T199P/C206S mutant. Biochemistry.

[29] Zheng J., Avvaru B.S., Tu C., McKenna R., Silverman D.N. (2008). Role of hydrophilic residues in proton transfer during catalysis by human carbonic anhydrase II. Biochemistry.

[30] Montgomery J.C., Venta P.J., Eddy R.L., Fukushima Y.S., Shows T.B., Tashian R.E. (1991). Characterization of the human gene for a newly discovered carbonic anhydrase, CA VII, and its localization to chromosome 16. Genomics.

[31] Earnhardt J.N., Qian M., Tu C., Lakkis M.M., Bergenhem N.C., Laipis P.J., Tashian R.E., Silverman D.N. (1998). The catalytic properties of murine carbonic anhydrase VII. Biochemistry.

[32] Di Fiore A., Truppo E., Supuran C.T., Alterio V., Dathan N., Bootorabi F., Parkkila S., Monti S.M., De Simone G. (2010). Crystal structure of the C183S/C217S mutant of human CA VII in complex with acetazolamide. Bioorg. Med. Chem. Lett..

[33] Del Giudice R., Monti D.M., Truppo E., Arciello A., Supuran C.T., De Simone G., Monti S.M. (2013). Human carbonic anhydrase VII protects cells from oxidative damage. Biol. Chem..

[34] Monti D.M., De Simone G., Langella E., Supuran C.T., Di Fiore A., Monti S.M. (2017). Insights into the role of reactive sulfhydryl groups of carbonic anhydrase III and VII during oxidative damage. J. Enzym. Inhib. Med. Chem..

[35] Truppo E., Supuran C.T., Sandomenico A., Vullo D., Innocenti A., Di Fiore A., Alterio V., De Simone G., Monti S.M. (2012). Carbonic anhydrase VII is S-glutathionylated without loss of catalytic activity and affinity for sulfonamide inhibitors. Bioorg. Med. Chem. Lett..

[36] Ruusuvuori E., Li H., Huttu K., Palva J.M., Smirnov S., Rivera C., Kaila K., Voipio J. (2004). Carbonic anhydrase isoform VII acts as a molecular switch in the development of synchronous gamma-frequency firing of hippocampal CA1 pyramidal cells. J. Neurosci..

[37] Rivera C., Voipio J., Kaila K. (2005). Two developmental switches in GABAergic signalling: The K^+^–Cl^−^ cotransporter KCC2 and carbonic anhydrase CA VII. J. Physiol..

[38] Bootorabi F., Janis J., Smith E., Waheed A., Kukkurainen S., Hytonen V., Valjakka J., Supuran C.T., Vullo D., Sly W.S. (2010). Analysis of a shortened form of human carbonic anhydrase VII expressed in vitro compared to the full-length enzyme. Biochimie.

[39] Asiedu M., Ossipov M.H., Kaila K., Price T.J. (2010). Acetazolamide and midazolam act synergistically to inhibit neuropathic pain. Pain.

[40] Avvaru B.S., Kim C.U., Sippel K.H., Gruner S.M., Agbandje-McKenna M., Silverman D.N., McKenna R. (2010). A short, strong hydrogen bond in the active site of human carbonic anhydrase II. Biochemistry.

[41] Fisher S.Z., Maupin C.M., Budayova-Spano M., Govindasamy L., Tu C., Agbandje-McKenna M., Silverman D.N., Voth G.A., McKenna R. (2007). Atomic crystal and molecular dynamics simulation structures of human carbonic anhydrase II: Insights into the proton transfer mechanism. Biochemistry.

[42] Hakansson K., Carlsson M., Svensson L.A., Liljas A. (1992). Structure of native and apo carbonic anhydrase II and structure of some of its anion-ligand complexes. J. Mol. Biol..

[43] Li H., Robertson A.D., Jensen J.H. (2005). Very fast empirical prediction and rationalization of protein pK_a_ values. Proteins.

[44] Olsson M.H., Sondergaard C.R., Rostkowski M., Jensen J.H. (2011). PROPKA3: Consistent treatment of internal and surface residues in empirical pK_a_ predictions. J. Chem. Theory Comput..

[45] Olsson M.H. (2011). Protein electrostatics and pK_a_ blind predictions; contribution from empirical predictions of internal ionizable residues. Proteins.

[46] Mikulski R., Domsic J.F., Ling G., Tu C., Robbins A.H., Silverman D.N., McKenna R. (2011). Structure and catalysis by carbonic anhydrase II: Role of active-site tryptophan 5. Arch. Biochem. Biophys..

[47] Mikulski R., West D., Sippel K.H., Avvaru B.S., Aggarwal M., Tu C., McKenna R., Silverman D.N. (2013). Water networks in fast proton transfer during catalysis by human carbonic anhydrase II. Biochemistry.

[48] Riccardi D., Konig P., Guo H., Cui Q. (2008). Proton transfer in carbonic anhydrase is controlled by electrostatics rather than the orientation of the acceptor. Biochemistry.

[49] Cudney R., Patel S., Weisgraber K., Newhouse Y., McPherson A. (1994). Screening and optimization strategies for macromolecular crystal growth. Acta Crystallogr. D Biol. Crystallogr..

[50] Jancarik J., Kim S.-H. (1991). Sparse matrix sampling: A screening method for crystallization of proteins. J. Appl. Crystallogr..

[51] Otwinowski Z., Minor W. (1997). Processing of X-ray diffraction data collected in oscillation mode. Methods Enzymol..

[52] Navaza J. (1994). Amore: An automated package for molecular replacement. Acta Crystallogr. Sect. A Found. Crystallogr..

[53] Brunger A.T., Adams P.D., Clore G.M., DeLano W.L., Gros P., Grosse-Kunstleve R.W., Jiang J.S., Kuszewski J., Nilges M., Pannu N.S. (1998). Crystallography & NMR system: A new software suite for macromolecular structure determination. Acta Crystallogr. D Biol. Crystallogr..

[54] Brunger A.T. (2007). Version 1.2 of the crystallography and NMR system. Nat. Protoc..

[55] Jones T.A., Zou J.Y., Cowan S.W., Kjeldgaard M. (1991). Improved methods for building protein models in electron density maps and the location of errors in these models. Acta Crystallogr. Sect. A Found. Crystallogr..

[56] Hooft R.W., Vriend G., Sander C., Abola E.E. (1996). Errors in protein structures. Nature.

[57] Laskowski R.A., MacArthur M.W., Moss D.S., Thornton J.M. (1993). Procheck: A program to check the stereochemical quality of protein structures. J. Appl. Crystallogr..

